# The specificity and genetic background of the rye (*Secale cereale* L.) tissue culture response

**DOI:** 10.1007/s00299-012-1342-9

**Published:** 2012-09-25

**Authors:** Małgorzata Targońska, Aneta Hromada-Judycka, Hanna Bolibok-Brągoszewska, Monika Rakoczy-Trojanowska

**Affiliations:** Department of Plant Genetics, Breeding and Biotechnology, Faculty of Horticulture and Landscape Architecture, Warsaw University of Life Sciences, SGGW, Nowoursynowska St 159, 02-776 Warsaw, Poland

**Keywords:** In vitro culture, Rye (*Secale cereale* L.), Tissue culture response

## Abstract

Rye is one of the most important crops in Eastern and Northern Europe. Despite the numerous beneficial features of rye, its annual production decreases successively which correlates with the lack of progress in its breeding compared with other cereals. Biotechnological methods could effectively improve the breeding of rye. However, their application is highly limited by the absence of an efficient procedure for plant regeneration in vitro, since rye is one of the most recalcitrant cereals with regard to the tissue culture response (TCR), and successful regeneration is highly dependent on genotype. Efforts to understand the genetic mechanisms controlling TCR of rye have elucidated some basic aspects, and several genes and genome regions controlling this trait have been identified. The aim of this review is to summarize the limited current knowledge of this topic.

## Introduction

Common rye (*Secale cereale* L.) is one of the most important cereals cultivated in Eastern and Northern Europe. The crop possesses a great number of advantages such as a unique nutritional value, winter hardiness and tolerance to environmental stresses as low temperatures, drought and poor soil conditions. Recently, nevertheless, a distinct reduction of cultivation area and yield of rye has been observed compared to other cereals. This is mainly caused by a relatively slow breeding progress connected, predominantly, with a high self-incompatibility and inbreeding depression. At present, the main task of rye breeding is the improvement of resistance to diseases (leaf rust, rhynchosporium, powdery mildew) and pre-harvest sprouting (the majority of cultivars are characterized by a medium value for these characters). Biotechnological methods, e.g., double haploid production, genetic transformation or selection of plants with beneficial somaclonal changes could effectively improve the breeding of rye. However, their application is limited by a lack of an efficient procedure of plant regeneration in vitro, as rye is one of the most recalcitrant cereals in regard to in vitro plant regeneration ability (Ma et al. 2003) that is, additionally, highly dependent on genotype (Linacero and Vazquez 1990; Rakoczy-Trojanowska and Malepszy 1993, 1995; Popelka and Altpeter 2001). The regeneration efficiencies from immature embryos of the best responding rye genotypes such as lines: L318, L20 or L4 are around 60.2, 59.6 and 52.9 %, respectively (Popelka and Altpeter 2001; Rakoczy-Trojanowska and Malepszy 1995), but in most of the genotypes it is not higher than a few percent. Similar observations have been found when other types of explants instead of immature embryos are used in experiments. The best forms, including spring rye, can regenerate to plant at a level of 30.6 green plants per 100 plated anthers (Immonen and Anttila 1999), whereas winter rye, such as line L318, <1 plant per 100 anthers (Rakoczy-Trojanowska et al. 1997). Similar relationships to factors influencing in vitro regeneration can be observed in other important crops. Wheat, as a member of the same family as rye, is also considered to be a recalcitrant crop according to in vitro culture response (Redway et al. 1990). However, regeneration of this plant is possible from different types of explants such as leaves, seeds, mature and immature embryos, shoot bases and root tips (Sarker and Biswas 2002). Moreover, plant regeneration from tissue culture can be predictable and stable when the appropriate genotype is used (Sears and Deckard 1982). The research of Mitić et al. (2006) concerning immature embryos from 96 different cultivars showed that there were genotypes whose ability to produce regenerating callus was over 70 % such as Donska polupatuljasta, UC 65680, NS 74/95 or Mexico 120. However, in the case of mature embryos of two spring varieties, regeneration efficiency was about 7 % (Rahman et al. 2008). In barley, despite plant regeneration from callus being controlled by several genes (Komatsuda et al. 1989; Mano et al. 1996) and there being variability in the type of in vitro response observed among genotypes (Bregitzer et al. 1998; Baillie et al. 1993), mature plants were successfully generated from different kind of explants. An example of highly regenerating genotype is the cultivar Hassan, in which about 80 % of calli obtained from mature embryos, regenerated into plants (Zapata et al. 2004). Plant regeneration from immature inflorescences is also effective with 34 plants per 64 explants with the variety Galan (Havrlentová et al. 2001). Research investigating callus induction and plant regeneration from immature embryos from different cultivars of triticale showed that the mean number of plant regeneration coefficient ranged between 9.7 for cultivar Gabo and 15.9 for the cultivar Wanad (Przetakiewicz et al. 2003). For microspore and anther cultures of wheat, barley or triticale, both the level of induction and regeneration are significantly higher than in the best forms of rye (Davies and Morton 1998; González and Jouve 2005; Castillo et al. 2000; Holme et al. 1999). Only some of spring lines and varieties of rye and *Secale vavilovii* respond at the average level of other forms of cereals (Guo and Pulli 2000; Rakoczy-Trojanowska et al. 1997).

Efforts to understand the genetic mechanisms controlling tissue culture response (TCR) have explored some basic aspects, and allowed the identification of several genes and genome regions controlling this trait (Bolibok et al. 2007; Hromada-Judycka et al. 2010; Gruszczynska and Rakoczy-Trojanowska 2011). The aim of this review is a presentation of the current, but still incomplete knowledge concerning this topic in rye.

## Specificity of rye TCR

Rye is a species characterized by a particularly poor TCR and in spite of many efforts (Lu et al. 1984; Zimny and Lörz 1989; Rakoczy-Trojanowska and Malepszy 1993, 1995; Rakoczy-Trojanowska et al. 1997), the efficiency of plant regeneration is still much lower than in other species, including cereals, regardless of the genotype, explant type and in vitro culture conditions (Lu et al. 1984; Krumbiegel-Schroeren et al. 1984; Linacero and Vazquez 1986; Rakoczy-Trojanowska and Malepszy 1993, 1995; Rakoczy-Trojanowska et al. 1997).

Many factors, both biotic (predominantly donor plant genotype, nature and developmental stage of explants) and abiotic (a broad range of culture conditions and interactions between them), have been tested to try to establish an optimal protocol for efficient and replicable in vitro regeneration of rye.

### Genotype

In rye, as in other plant species, the genotype of the donor plant is one of the most important factors influencing TCR (Krumbiegel-Schroeren et al. 1984; Linacero and Vazquez 1986; Rakoczy-Trojanowska and Malepszy 1993, 1995; Rakoczy-Trojanowska et al. 1997; Popelka and Altpeter 2001; Ma and Pulli 2004). In general, the efficiency of rye TCR is low, usually around 20–30 %, although several genotypes respond much better, e.g., the inbred lines L318 and L22 (Rakoczy-Trojanowska and Malepszy 1993, 1995; Rakoczy-Trojanowska et al. 1997; Popelka and Altpeter 2001), wild species *S. africanum* and *S. kuprianovii* (Rybczyński and Zduńczyk 1986), interspecific hybrids *S. cereale* × *S. vavilovii* (Flehinghaus et al. 1991; Flehinghaus-Roux et al. 1995), some spring cultivars—Florida 401, Jo02 (Lu et al. 1984; Immonen and Anttila 1999; Ma and Pulli 2004; Guo and Pulli 2000); and some winter cultivars—Zulpan, Amilo, Jussi (Guo and Pulli 2000; Ma et al. 2004). However, apart from a few individual cases, the agronomic value and/or usefulness for breeding of these more compliant genotypes is rather low.

**Fig. 1:**
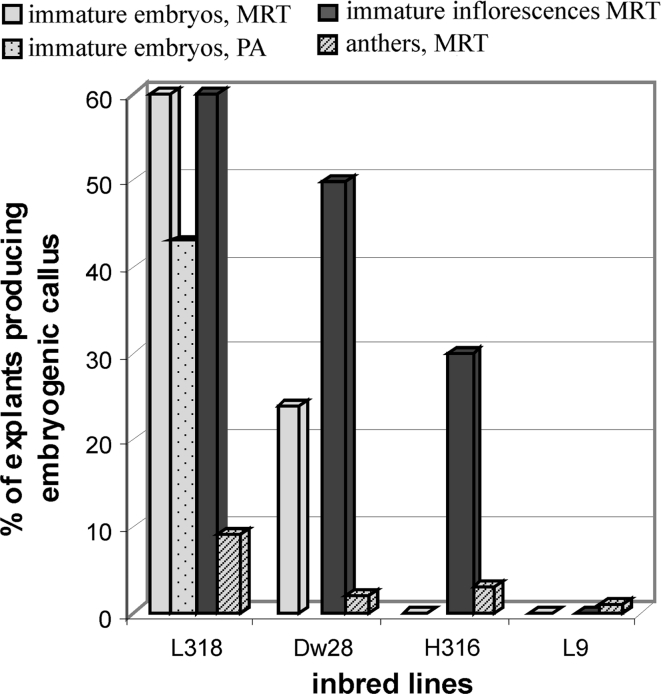
TCR of selected rye inbred lines producing embryogenic callus from different explants. Based on Rakoczy-Trojanowska and Malepszy (1993, 1995), Rakoczy-Trojanowska et al. (1997) (MRT) and Popelka and Altpeter (2001) (PA)

Among rye forms studied with respect to TCR, several lines and/or cultivars have been characterized as “universally responding” (i.e., the level of regeneration in vitro is similar regardless of the culture or explant type), whereas the majority of those that show a response are “differentially responding” (i.e., the level of regeneration in vitro depends on medium composition, explant type, other culture conditions and interactions between these factors). Examples of “universally responding” forms are line L318, which is classified as a positively responding genotype, and line L9, which is a non-responding one (Fig. 1).

However, most of the donor genotypes tested show a response to tissue culture only with one type of explant and/or under strictly defined culture conditions. For example, plants of inbred line H363 could be regenerated from immature inflorescences at an efficiency of over 75 % (Rakoczy-Trojanowska and Malepszy 1993), but completely failed to respond in the case of immature embryo (Rakoczy-Trojanowska and Malepszy 1995) or anther cultures (Rakoczy-Trojanowska et al. 1997). In contrast, no plants could be regenerated from immature inflorescences of line H316, but the regeneration efficiency of immature embryos was as high as 30 % (Rakoczy-Trojanowska and Malepszy 1993, 1995).

### Explant type

A number of different somatic explants of rye have been used for callus induction and/or shoot/root/plant regeneration: leaf fragments (Rybczyński 1980; Linacero and Vazquez 1986), immature embryos and/or their fragments (Rybczyński 1979; Rybczyński and Zduńczyk 1986; Eapen and Rao 1982; Rakoczy-Trojanowska and Malepszy 1995), mature embryos (Rybczynski 1980; Ward and Jordan 2001), and immature inflorescences and/or their fragments (Rybczynski et al. 1980; Linacero and Vazquez 1990; Rakoczy-Trojanowska and Malepszy 1993; Eapen and Rao 1985; Barro et al. 1999). The TCR has been most frequently observed with immature embryos and immature inflorescences, although the efficiency of plant regeneration is significantly influenced by their stage of development. Lu et al. (1984) and Zimny and Lörz (1989) showed that the developmental stage of immature embryos was the critical factor affecting regeneration efficiency, with the best results (up to 100 % of explants regenerating plants) obtained with embryos in the late spherical coleoptile stage. In the case of immature inflorescences, optimal results have mainly been achieved with 0.5–2 cm long explants (Rybczynski 1980; Rybczynski et al. 1980; Rakoczy-Trojanowska and Malepszy 1993; Barro et al. 1998).

Both anthers and microspores have been successfully employed for haploid production (Flehinghaus-Roux et al. 1995; Rakoczy-Trojanowska et al. 1997; Guo and Pulli 2000; Ma et al. 2004), but the level of green plant regeneration was relatively low.

Little success has been achieved using suspension and protoplast cultures of rye. Ma et al. (2003) developed a method for embryogenic callus induction and fertile plant regeneration from suspension cell-derived rye protoplasts, but only 7 % of the embryogenic calli transferred to solid MS medium produced green shoots.

There have also been attempts to obtain rye cell aggregates from suspension cultures to act as material for plant genetic modification (Mottley and Sybenga, 1988). However, despite the fact that microcolonies transferred to agar medium containing 2,4-D (2.5 mg/l) produced normal callus, only roots were formed after this callus was transferred to hormone-free medium. On the other hand, it was also shown that culture in aggregates did not affect the growth or limit the regeneration properties of the cells (Mottley and Sybenga 1988).

### Culture conditions

Several basal media have been tested in order to optimize the regeneration process in tissue cultures of rye: MS (Rybczynski 1980; Rybczynski et al. 1980; Zimny and Lörz 1989; Lu et al. 1984), N6, CC-10 and B5 (Zimny and Lörz 1989), and SH (Rybczynski 1980). Medium SH has also been employed with the addition of different plant growth regulators (PGR) in various combinations and concentrations (Rybczynski 1980; Rybczynski et al. 1980; Zimny and Lörz 1989; Lu et al. 1984), and organic supplements such as coconut water (Zimny and Lörz 1989; Lu et al. 1984) or casein hydrolysate (Lu et al. 1984). The results of around 30 years of study indicate that MS supplemented with 2,4-D or Dicamba (1–3 mg/dm^3^) and sucrose (30 g/dm^3^) is the best induction medium for somatic tissues, whereas most efficient plant regeneration is promoted by MS (or half strength MS) with IAA (usually 2 mg/l) or lacking PGR. On the other hand, Zimny and Lörz (1989) found CC-10 (with 30 μM Dicamba) to be the best medium for callus production from immature embryos (33–47 % efficiency of somatic embryogenesis).

### Interactions between factors

Interactions between biotic and abiotic factors that influence the efficiency of rye TCR have been examined in a few studies. The findings of a series of detailed experiments carried out by Popelka and Altpeter (2001) led to the development of genetic-specific tissue culture protocols to maximize plant regeneration in vitro. They found that the genotype and the sources of carbohydrate and auxin influenced callus induction and maintenance, the germination of explants and the regeneration response. Separate genotypes differed in the callus response according to the basic salt composition of the medium, the gelling agent employed, CuSO_4_ complementation, the media sterilization procedure and illumination. A similar study conducted by Zimny and Lörz (1989) demonstrated the importance of interactions between genotype, medium composition and the developmental stage of explants. Through “step by step” optimization, they defined optimal culture conditions that produced 90–100 % efficiency of somatic embryogenesis and a high number of regenerated plants (Zimny and Lörz 1989). The aforementioned studies showed that genotype-specific adjustment of many components and factors are essential in order to achieve high regeneration potential in rye. This information also provided the basis for the development of a protocol for the genetic transformation of rye (Popelka and Altpeter 2001).

## Genetic control of rye TCR

### Mendelian analysis

Genetic analysis at the Mendelian level, performed in F_1_, F_2_ and F_3_ generations obtained from crosses between selected inbred lines (DW28, H363, L318, D855, H32, Pw330, L9, L29 L299 and H316) that differ in their TCR showed that the in vitro response of immature embryos and immature inflorescences is controlled by a complex, polygenic system with various gene interactions, and that the plant regeneration ability is a recessive trait (Rakoczy-Trojanowska and Malepszy 1993, 1995). For both explant types, embryogenic callus production, and plant and root regeneration appear to be determined by recessive genes or suppressed by two dominant non-allelic complementary genes, whereas the reduced ability to produce non-embryogenic callus is most probably controlled by dominant genes. The lack of response was shown to be controlled by at least two interacting genes. The main difference between these two explant types is apparently caused by a heterosis effect, which positively influences embryogenic callus production and plant regeneration exclusively in immature embryos (Rakoczy-Trojanowska and Malepszy 1995). Heterosis of donor plants was also found to promote androgenic plant regeneration from rye anthers (Flehinghaus et al. 1991; Flehinghaus-Roux et al. 1995).

### Effect of chromosomes

Three types of plant materials have been employed in cytogenetic analyses designed to type rye chromosomes carrying genes influencing TCR of various explants: wheat-rye addition lines (Lazar et al. 1987; Martinez et al. 1994), wheat-rye substitution lines (Pershina et al. 2003; Dobrovolskaya et al. 2003) and recombinant wheat-rye lines carrying segments of 1RS chromosome—1RS/1BL (Langridge et al. 1991). These studies examined the chromosomal location of positive and negative factors influencing TCR of immature embryos and anthers, and showed that both TCR of different explants and individual parameters (embryogenesis induction, total plant regeneration, green plant regeneration in the case of anther culture) are generally controlled by different genetic mechanisms (Table 1). However, the significance and universality of these findings for rye biotechnology is rather limited because (1) they in fact elucidated the TCR of wheat rather than of rye, (2) the genetic factors that enhance and reduce TCR are spread across all rye chromosomes (which is not surprising, bearing in mind the complexity of TCR), (3) these studies described the effects of rye chromosomes coming from certain forms in a defined wheat genetic background, and (4) the results are often contradictory, e.g., the study of Pershina et al. (2003) investigated the consequence of the substitution wheat cv. Saratovskaya 29/rye cv. Onokhoiskaya, while the donor materials used by Langridge et al. (1991) was from wheat cv. Chinese Spring with a translocated fragment of rye chromosome 1RS from cv. Imperial. In addition, Lazar et al. (1987) showed that chromosome 6R contains factors promoting callus production and plant regeneration from immature embryos, while Pershina et al. (2003) found negatively acting genes in this chromosome. Similarly, chromosome 1R has been identified as the location of both positive (Dobrovolskaya et al. 2003) and negative (Martinez et al. 1994) factors controlling in vitro androgenesis in rye. These inconsistencies might be explained by the complexity of TCR.

**Table 1:** Chromosomal location and effects of factors influencing TCR of immature embryos and anthers

Explant type	Rye chromosomes encoding factors affecting TCR		Type of material used for analyses	References
	Positively/trait	Negatively/trait		
Immature embryos	6R, 7R/ECF	−	Wheat-rye addition lines	Lazar et al. (1987)
	1R/EC, PR	−	Recombinant wheat-rye lines (1RS/1BL)	Langridge et al. (1991)
	2R, 3R/ECF	2R/R 6R, 1R/PR	Wheat-rye substitution lines	Pershina et al. (2003)
Anthers	3R, 4R	5R, 1R, 3R	Wheat-rye addition lines	Martinez et al. (1994)
	4R/ECF, PR	−	Wheat-rye addition lines	Lazar et al. (1987)
	1R	5R	Wheat-rye substitution lines	Dobrovolskaya et al. (2003)

*ECF* embryogenic callus formation, *RR* root regeneration, *PR* plant regeneration

### Molecular analysis

The application of molecular methods has both extended existing knowledge about rye TCR and also verified the results of earlier research employing Mendelian and cytogenetic analyses (Rakoczy-Trojanowska and Malepszy 1993, 1995). So far, three approaches have been used to elucidate different aspects of rye TCR: the identification of QTLs (Quantitative Trait Loci), gene orthologs and GDDSC (Genetically Directed Differential Subtraction Chain)-derived sequences controlling callus induction and somatic embryogenesis (SE).

#### QTLs for rye TCR

To identify QTLs for TCR in rye, a RIL (Recombinant Inbred Lines) mapping population was developed from the cross L318 × L9. A total of 102 RILs (F_5_ and F_6_) were used for phenotypic evaluation. A QTL analysis based on four parameters describing the reaction of immature inflorescences and immature embryos resulted in the identification of nine putative QTLs controlling rye TCR (Table 2). These were located on chromosomes 1R, 4R (two QTLs), 5R (two QTLs), 6R (two QTLs) and 7R (two QTLs). The highest number of QTLs (four) was identified for the percentage of immature embryos producing somatic embryos (ESE). The proportion of total phenotypic variation explained by individual QTLs ranged from 10.8 to 41.6 %. The value of variance for the model considering the detected QTLs for ESE together (69.1 %) indicates that the major loci influencing the in vitro response of immature rye embryos in the studied population have been identified.

**Table 2:** Characteristics of QTLs controlling rye TCR (based on Bolibok et al. 2007)

Chromosome	Trait	QTL	LOD score	Weight	Variance explained (%)
1R	ESE	*ese*-*1*	3.78	16.929	28.4
4R	ESE	*ese*-*2*	6.0	2.33	18.2
	ICI	*ici*-*7*	2.32	8.029	11.4
5R	ECI	*eci*-*1*	3.59	−31.309	20.8
	ESE	*ese*-*3*	2.55	17.453	24.2
6R	ECI	*eci*-*2*	3.23	−31.223	22.1
	ESE	*ese*-*4*	3.64	10.581	41.6
7R	ICI	*ici*-*2*	3.67	11.008	20.6
	ISE	*ise*-*2*	2.40	−9.203	10.8

*ECI* % of immature embryos producing callus, *ICI* % of immature inflorescences producing callus, *ISE* % of immature inflorescences forming embryogenic callus, *ESE* % of immature embryos forming embryogenic callus

#### TCR-connected genes of rye

Several genes responsible for callus induction (Nishimura et al. 2005) and SE have been well characterized in plants (Chugh and Khurana 2002; Ikeda et al. 2006). Four genes that have been studied as candidates controlling TCR in rye are the three crucial genes *SERK* (*Somatic Embryogenesis Receptor*-*Like Kinase*), *LEC1* (*Leafy Cotyledon 1*) and *NiR* (*Nitrate Reductase*), plus *Vp1* (*Viviparous 1*), a gene not previously investigated with respect to TCR (Gruszczyńska and Rakoczy-Trojanowska 2011). *SERK* encodes a RLK (*Receptor*-*Like Kinase*) protein and it has been shown to play an important role during somatic embryogenesis induction in many plants, e.g., carrot *Dactylis glomerata* and *Arabidopsis thaliana* (Schmidt et al. 1997; Somleva et al. 2000; Hecht et al. 2001). *LEC1* controls different aspects of embryo development and it is considered as the main regulator of embryogenesis in *A. thaliana*. During early embryogenesis (both zygotic and somatic), *LEC1* is necessary to maintain the embryogenicity of cells (Meinke 1992; West et al. 1994; Lotan et al. 1998). In late embryogenesis, *LEC1* is involved in seed maturation (West et al. 1994; Meinke et al. 1994; Parcy et al. 1997; Vicient et al. 2000). *VP1* is the main regulator of late embryogenesis in maize and it has two functions: (1) to regulate the activation of genes taking part in embryo maturation, and (2) to inhibit the expression of genes coding for hydrolases in the course of cob development and maturation (McKibbin et al. 2002). *NiR* encodes ferredoxin-nitrate reductase, a key enzyme in assimilation of the nitrogen source nitrate. This enzyme is not directly involved in SE, but it enables the induction of callus formation from immature embryos of rice and cotton, and, consequently, plant regeneration, since it catalyzes the reduction of nitrite, which has a toxic effect on cell growth, to ammonium (Nishimura et al. 2005; Han et al. 2010). The transcript levels of rye orthologs of these genes were measured during the subsequent in vitro culture periods, and the sites of expression were localized in zygotic embryos. Their expression profiles indicated that the function of these genes is correlated with TCR in rye. During the culture of immature embryos of line L9, increased levels of the rye *SERK* ortholog were observed at most stages. The suppression of *ScSERK* expression appeared to start after the induction of somatic embryogenesis and continued until plant regeneration. It is possible that the homologs of *LEC1* and *VP1* in rye act in a complimentary manner and have a negative effect on the production of embryogenic callus. The expression of the *NiR* homolog during in vitro culture confirmed its importance in the process of plant regeneration.

#### GDDSC-derived sequences and their likely role in rye TCR

**Fig. 2:**
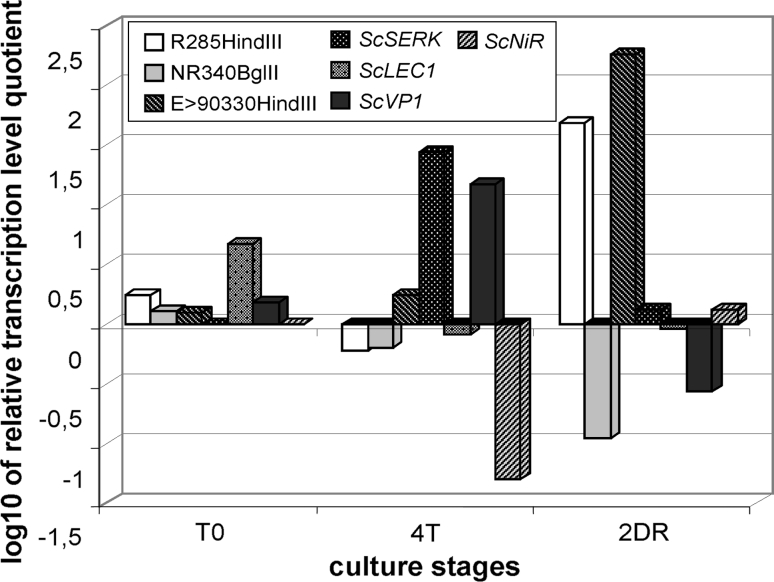
Logarithmic plot of the relative transcription level quotients of selected GDDSC products and Sc genes between the L318 and L9 rye lines. Values: >0—expression level higher in line L318; <0—expression level higher in line L9. T0—immature embryos, T4—tissue collected after 4 weeks on induction medium, 2DR—tissue collected after 2 days on regeneration medium (base on data from Hromada-Judycka et al. 2010; Gruszczyńska and Rakoczy-Trojanowska 2011)

The molecular mechanisms controlling rye TCR were further examined by the application of GDDSC, a method that permits the isolation of specific sequences from DNA bulks that differ with respect to the investigated trait and also generates markers tightly linked to this trait (Przybecki et al. 2004). Two pairs of bulks, composed of DNA isolated from recombinant inbred lines, were created: the first comprised DNA from RILs capable (R) and incapable (NR) of plant regeneration, and the second, DNA from RILs capable (*E* > 90) and incapable (*E* < 25) of embryogenic callus formation. The application of this method generated 47 unique sequences for characterization (Hromada-Judycka et al. 2008, 2010; Siedlecka et al. 2011; Hromada-Judycka 2011; unpublished). Most of the GDDSCs were similar to the sequences flanking genes involved in different metabolic processes, such as stress responses, amino acid transport and fatty acids synthesis. Three resembled sequences that are in close proximity to the genes encoding *CBF10* (CRT/DRE binding factor), amino acid permease and acetyl-CoA carboxylase, respectively, which indicates that they may play some regulatory function in the transcription of these genes. The gene *CBF10* together with its potential regulating factors are particularly interesting, since increased expression of CRT/DRE-related genes is usually associated with osmotic stress that is present under tissue culture conditions. Real-Time RT-PCR analysis of GDDSCDSs selected by bioinformatic analysis showed their diverse expression at different stages in the culture of tissues from the well responding inbred line L318 and the non-reactive line L9. The expression profiles of the majority of isolated sequences were in agreement with the subtraction direction, i.e., the transcript levels of *R* and *E* > 90 sequences were higher in line L318 than in line L9 (at least in the critical tissue culture stages). Nevertheless, some of the GDDSCDSs showed the opposite pattern of transcription, or their expression profiles were more complicated, being in agreement with the subtraction direction only at some culture stages. A similar phenomenon was observed in the case of SE-related rye orthologs: enhanced expression of the *ScSERK*, *ScVP1*, *ScLEC1* and *ScNiR* transcripts in the positively responding line L318 was limited to the regeneration phase (Fig. 2).

## Mendelian versus molecular analysis

**Fig. 3:**
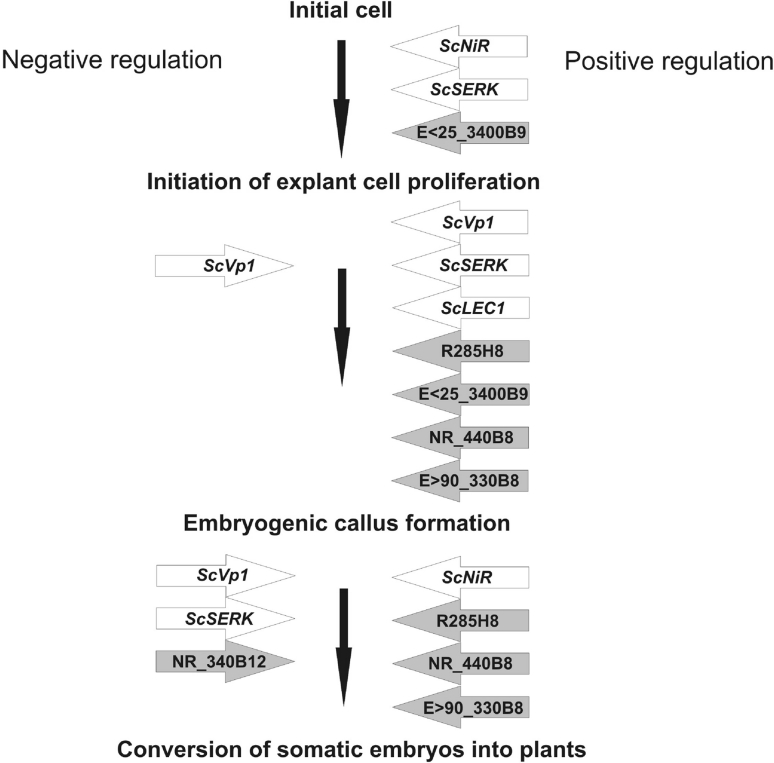
Gene orthologs and GDDSC products that positively or negatively affect the subsequent steps of rye TCR

In general, the results of molecular studies have confirmed the conclusions drawn from Mendelian and cytogenetic analyses, and show that at least two processes in rye, namely callus production and plant regeneration, are recessive traits regulated in a complex manner. For example, orthologs of the *LEC1* and *VP1* genes may interact in a complementary manner to perform the role of negative regulators of embryogenic callus development distinguished by Mendelian analyses. In addition, *ScVP1* seems to suppress *ScSERK*, which positively regulates the initiation of processes leading to somatic embryogenesis (Gruszczyńska and Rakoczy-Trojanowska 2011). Similarly, amongst GDDSC products isolated by Hromada-Judycka et al. (2008, 2010), at least some (e.g., NR_340Bl8) appear to act as negative regulators of plant regeneration (Fig. 3).

The results of both cytogenetic and QTL analyses suggest that the factors influencing rye TCR are spread across multiple chromosomes: 1R, 3R, 5R 6R, 7R according to cytogenetic data (Lazar et al. 1987; Martinez et al. 1994), and 1R, 3R, 4R, 5R, 6R, 7R based on molecular data (Grosse et al. 1996; Bolibok et al. 2007). However, precise comparisons are impossible due to the lack of common markers. Thus, it is uncertain whether the results of the aforementioned studies implicate corresponding regions of rye chromosomes, or different regions within the same chromosome. Nevertheless, it is clear that TCR in rye is a complex and polygenic trait.

## Concluding remarks

Although knowledge about the genetic control of TCR in rye is still limited and rather fragmentary, and the use of tissue culture for breeding and genetic manipulation remains very difficult, the results of investigations conducted over the last decade have uncovered details of some of the mechanisms involved. Several genes and genome fragments that play an important role in the regulation of TCR have been discovered. Some of these may serve as the source of molecular markers for the selection of positive genotypes, particularly the GDDSC products *R*_285H8 and *E* > 90_330B8, as their expression level was found to be considerably higher in almost all tissue culture stages of the positively responding line L318. Conversely, markers based on the GDDSC product NR_340Bl8, and the *ScVP1* and *ScLEC1* genes may assist in the selection of negatively responding forms. The rapid development of molecular techniques such as NGS should significantly accelerate work in this field in the near future.
